# Temporal Parameters of Spontaneous Speech as Early Indicators of Alcohol-Related Cognitive Impairment

**DOI:** 10.3390/jcm15031092

**Published:** 2026-01-30

**Authors:** Fanni Fruzsina Farkas, Ildikó Hoffmann, Otília Bagi, Janka Gajdics, Bálint Andó, Gábor Gosztolya, Ildikó Kovács, Bence András Lázár, János Kálmán

**Affiliations:** 1 Department of Psychiatry, Albert Szent-Györgyi Medical School, University of Szeged, 6720 Szeged, Hungary; 2 HUN-REN Research Centre for Hungarian Linguistics, 1068 Budapest, Hungary; 3 HUN-REN-SZTE Research Group on Artificial Intelligence, 6720 Szeged, Hungary; 4 Institute of Informatics, University of Szeged, 6720 Szeged, Hungary

**Keywords:** alcohol dependence syndrome, alcohol-related cognitive impairment, spontaneous speech, temporal speech parameters, S-GAP Test, early recognition, automatic speech recognition

## Abstract

**Background/Objectives**: Most patients with alcohol use disorder (AUD) suffer from mild cognitive decline, which does not meet the diagnostic criteria of the severe form of alcohol-related cognitive impairment (ARCI). ARCI is associated with executive abnormalities in addictive behaviors and therefore influences relapse and daily functioning. Abnormalities in speech production reflect cognitive disturbances. The aim of this study was to examine the temporal speech parameters (TSPs) in ARCI. **Methods**: The TSPs were measured with the S-GAP Test^®^ on 34 AUD patients with intact cognitive functions and 31 age- and gender-matched control participants. **Results**: Ten out of fifteen parameters of TSPs were significantly different between the AUD and healthy groups. Speech tempo and the total pause duration rate have significant classification potential. **Conclusions**: Our exploratory study revealed that filled pause-related temporal parameters appear to be particularly altered in ARCI and indicated that marked TSP alterations could serve as early indicators of cognitive deficits.

## 1. Introduction

Alcohol use disorder (AUD) as a chronic mental disorder is one of the most common forms of addictive behavior. It has been indicated that AUD was seventh on the list of leading risk factors for premature death and disability globally in 2016 [GBD, WHO]. During the course of AUD, various short-, mid- and long-term complications can be detected [1,2]. Besides the acute consequences of alcohol dependence such as alcohol withdrawal syndrome and delirium tremens, several studies have revealed the importance of neurocognitive function impairment [3,4,5,6].

The incidence of cognitive decline among alcohol-dependent individuals is approximately 40% [6]. Cognitive decline is an important contributor to relapse risk [7,8]. Although the most severe form of alcohol-related cognitive impairment (ARCI) is Korsakoff syndrome, other syndromes like mild cognitive decline, which does not meet the criteria of Korsakoff syndrome, play a pivotal role in the day-to-day functioning of patients with AUD [9,10,11,12,13,14].

ARCI includes abnormalities in visuospatial processing [6], working memory [15,16], semantic memory [17], free recall [18], executive functions [19,20,21], verbal fluency [22] and prospective memory [23]. Significant differences have been found in mental balance, attention and concentration, remote memory, delayed recall, immediate recall and visual retention [15]. These cognitive abnormalities could be improved during an abstinence period [6].

Cognitive and language processes are closely related. Research has extensively explored the impact of alcohol dependence on cognitive abilities, but fewer studies have specifically investigated the effects of long-term alcohol use on language processes (e.g., [24,25,26,27]). In an investigation of the speech comprehension of alcohol-dependent individuals, a significant deficit was found [25]. On the other hand, regarding speech production, few studies have examined semantic and phonemic fluency alterations in alcohol dependence [20,24,27].

In our previous research, we found that cognitive impairment can be detected at an early stage by examining TSPs [28,29,30,31]. ARCI is characteristically prevalent in AUD. However, it remains to be elucidated whether expressive language-related parameters are affected in AUD. Therefore, the main goal of this study was to investigate the TSPs in AUD.

## 2. Materials and Methods

### 2.1. Participants

Inpatients (*n* = 34; 25 male, 6 female; mean age (SD): 48.35 (SD = 9.39)) admitted with a principal diagnosis of AUD/alcohol dependence syndrome (F10.20) according to the International Classification of Diseases, Tenth Revision (ICD-10) at the Department of Psychiatry, University of Szeged, Hungary, between 1 July 2022 and 30 June 2023 were enrolled in this study.

The major inclusion criteria were the following: a diagnosis of AUD (F10.20); age between 18 and 60 years old; and a minimum of 7 days of alcohol abstinence.

The exclusion criteria were as follows: symptoms of alcohol withdrawal syndrome (up to seven points on the Clinical Institute Withdrawal Assessment of Alcohol Scale Revised (CIWA-Ar) [1,32,33]; clinically significant cognitive decline (Mini-Mental State Examination (MMSE) [34]; standardized in Hungarian by Janka et al. [35], score below 26 points); clinically significant somatic and/or neurological disorders; diagnosis of benzodiazepine use disorder; or pharmacological treatment affecting cognitive functioning.

Participants in the control group were chosen by convenience sampling from recruited healthy volunteers and were matched in age (SD: 50.71) and gender (20 male, 10 female). Patients with clinically significant cognitive decline (MMSE score below 26 points), problematic alcohol use (up to 7 points on the Alcohol Use Disorders Identification Test [AUDIT]), clinically significant somatic and/or neurological disorders, or a diagnosis of any other psychiatric disorder were excluded.

The AUD patients and cognitively healthy controls were native speakers of the Hungarian language.

This study was conducted in accordance with the principles of the Declaration of Helsinki. This study was approved by the Human Investigation Review Board, University of Szeged (ethical approval number: 109/2022-SZTE RKEB, 5217).

### 2.2. Neuropsychological Assessment

The assessment was performed after the eighth day of alcohol abstinence. The CIWA-Ar served as a measure of the severity of alcohol withdrawal. For screening and identifying hazardous and harmful drinkers and people with alcohol dependence, the AUDIT [36,37] was used, with a cut-off score of 7 points. In the statistical analyses the AUDIT subscales were also considered: AUDIT-C = Consumption subscale, AUDIT-D = Dependence subscale, AUDIT-HE = Harmful Effects subscale. Basic cognitive skills were assessed with the Mini-Mental State Examination (MMSE). This was followed by collecting spontaneous speech samples with the S-GAP Test^®^. Participants were asked to talk about their previous day in as much detail as possible for a minimum of 1 min. During speech collection, the investigator did not speak. An Olympus VN-541PC voice recorder device was used for speech sample collection. The total time of examination was 30–45 min.

### 2.3. Speech Analysis

Automatic speech recognition (ASR) technology was applied with recognition at the phonetic level (instead of the standard word-level speech recognition used in, e.g., speech-to-text systems), where the output of the ASR system consisted of phoneme-like units. These units, in addition to the phonemes of the Hungarian language, also included special tokens like silence or filled pauses as special phones. The ASR system employed standard speech recognition techniques. The Hidden Markov Model Tool Kit (HTK) [38] was modified to allow the use of a Hidden Markov Model/Deep Neural Network (DNN) hybrid setup [39] by incorporating an acoustic DNN model. The technical parameters of the acoustic DNN model were as follows: the acoustic features were 40 raw Mel filter bank energy values along with log-energy and the first- and second-order derivatives (‘FBANK + Δ + ΔΔ’), which resulted in 123 acoustic features overall.

Training and evaluation were performed on a 150 ms wide sliding window with a standard 10 ms step size. The acoustic DNN consisted of five fully connected hidden layers, each containing 1024 rectified linear neurons [40], whereas the final layer had 911 neurons, equal to the number of phonetic states.

The DNN acoustic model was trained on a subset of approximately 60 h of recordings from a specific Hungarian spoken language database (BEA corpus), which contains three large corpora with more than 500 h of spoken language. The BEA corpus was created by the Hungarian Research Centre for Linguistics [41]

The recordings from the BEA corpus were noise-augmented to better suit noisy recording conditions during recognition (which can be expected in speech processing applications). That is, noise, background speech and reverberation were added to the recordings of the BEA corpus before training, increasing the amount of training material to 240 h.

This ASR framework was utilized to perform speech recognition on the phonetic level; therefore, it provided a time-aligned token sequence for each recording: it supplied the hypothesis of the sequence of tokens (i.e., phoneme-like units uttered), along with the starting and ending time points for each token.

Table 1 summarizes the list and definitions of the 15 temporal parameters of spontaneous speech.

Since we investigated the presence of both silent and filled pauses, these parameters were calculated in three variations: for silent pauses only, for filled pauses only, and for both silent and filled pauses. This process led to 15 TSPs.

### 2.4. Statistical Analysis

First, descriptive statistical analyses were used for demographic variables, and the Shapiro–Wilk test was then performed to test normality. For normally distributed variables, independent sample *t*-tests were used, and for variables that did not follow a normal distribution, the Mann–Whitney U test was performed. Differences were considered significant if the *p*-value was ≤0.05. Two-tailed hypotheses were used.

Receiver operating characteristic (ROC) analysis was used to test the classification potential of TSPs, and to determine possible cut-off points for them. For the ROC analysis, a correlation matrix was used to determine the highly correlating TSPs: when the correlation coefficient was above 0.9, the most relevant variable was chosen to be analyzed. The remaining variables were tested with ROC analysis. The remaining variables were the total pause occurrence rate, filled pause frequency, total pause duration rate, total pause average length, speech tempo and silence frequency. In the case of 5 variables, the correlation coefficient was below 0.9: the silent pause occurrence rate, total pause occurrence rate, silent pause frequency, total pause frequency and filled pause average duration. The mentioned variables were tested with ROC analysis. We excluded the following variables: the silent pause duration rate, filled pause occurrence rate, filled pause duration rate, total pause duration rate and silent pause average duration. Using the Bonferroni correction, the classification potential was considered significant if the *p*-value was equal to or below 0.0045.

Statistical analyses were performed using IBM SPSS 24.0 (SPSS Inc., Chicago, IL, USA).

## 3. Results

### 3.1. Descriptive Statistics

The clinical characteristics of the participants are summarized in Table 2. A total of 18 female (27.7%) and 47 male (72.3%) participants were in the sample. In the AUD group, there were 7 female (20.6%) and 27 male (79.4%) patients, while in the control group, there were 11 female (35.5%) and 20 male patients (64.5%), but this difference was not significant (χ^2^(1, 65) = 1.797, *p* = 0.180). The mean age of the AUD group was 48.35 years (SD = 9.39), while the mean age of the control group was 50.71 years (SD = 9.795), but this difference was not significant either (*t*(63) = 0.990, *p* = 0.326).

### 3.2. Group Comparison of Psychometric Test Results

The Shapiro–Wilk test of normality was conducted to determine whether the data was normally distributed. The results indicated rejection of the null hypothesis and that the data was not normally distributed. Therefore, Mann–Whitney U tests were used to compare the psychometric test results of the AUD and control groups.

The median of the AUDIT score in the AUD group was significantly higher than that in the control group. Moreover, the medians of the AUDIT-C, AUDIT-D and AUDIT-HE subscales were also significantly higher in the AUD group (see Table 2).

There was no significant difference between the groups in the MMSE total score (z = −0.752, *p* = 0.452).

### 3.3. Group Comparison of Temporal Speech Parameters

The Kolmogorov–Smirnov test of normality was used to test the distribution of the TSPs; all of them were normally distributed. Ten of the fifteen linguistic parameters showed significant differences between the two groups. Speech tempo was significantly higher in the control group, and silent pause frequency was also higher in the control group. The filled pause occurrence rate, total pause occurrence rate, silent pause duration rate, filled pause duration rate, total pause duration rate, filled pause frequency, silent pause average duration and total pause average duration were higher in the AUD group. The speech tempo, filled pause occurrence rate, total pause duration rate and silent pause average duration had large effect sizes according to Cohen’s d; the other significantly differing TSPs had medium effect sizes. The results of the statistical comparisons can be seen in Table 3.

### 3.4. Receiver Operating Characteristic Analysis of Temporal Speech Parameters

We used ROC analysis to measure the classification potential of TSPs for detecting AUD. The AUDIT cut-off points were used to determine the severity categories of alcohol use problems: 20 points or more indicated AUD. A correlation matrix was used to find the highly correlating TSPs: the variables where the correlation coefficient was higher than 0.9 were examined, and the most relevant ones were kept in the ROC analysis. After the removal of the strongly related variables, 11 speech parameters remained in the ROC analysis. We used a Bonferroni correction on the level of significance to further lessen the probability of Type I error. According to this, the test was significant if *p* ≤ 0.0045.

**Figure 1:**
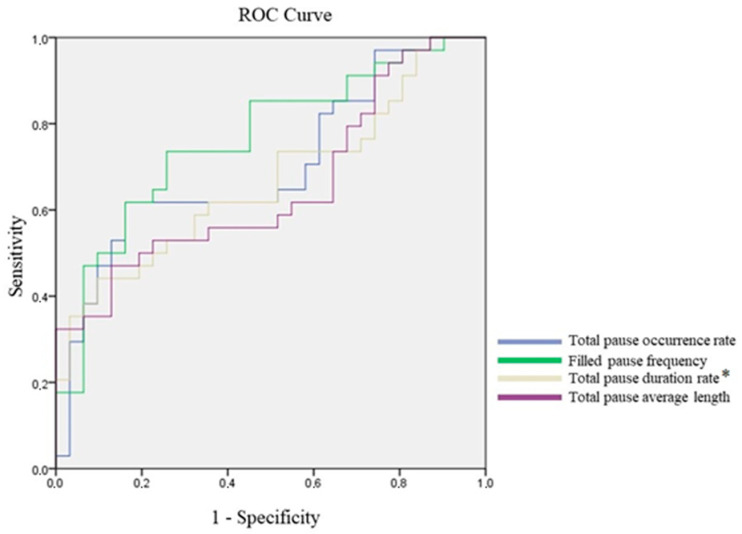
ROC analysis of temporal speech parameters with classification potential where higher value indicates **higher** AUD potential; * = significant after Bonferroni correction.

**Figure 2:**
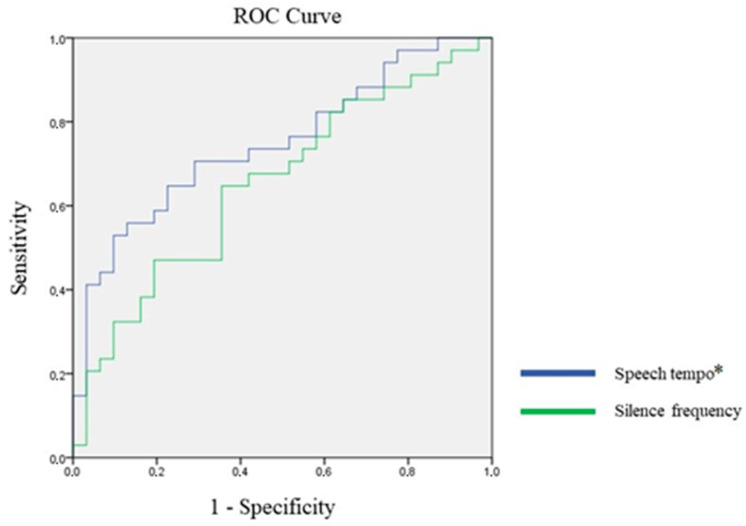
ROC analysis of temporal speech parameters with classification potential where higher value indicates **lower** AUD potential; * = significant after Bonferroni correction.

Without the Bonferroni correction, six TSPs had significant classification potential. In case of the total pause occurrence rate, filled pause frequency, total pause duration rate and total pause average length, higher values predicted a higher likelihood of AUD (Figure 1), while lower values of speech tempo and silence frequency predicted a higher likelihood of AUD (Figure 2). After the Bonferroni correction, the total pause duration rate and speech tempo continued to have significant classification potential. Using the Youden Index of the coordinates of the curve, we identified the cut-off point for the total pause duration rate as 37.746, with sensitivity of 73.5% and specificity of 74.2%. Regarding speech tempo, the cut-off point was 7.982, with sensitivity of 52.9% and specificity of 90.3%. The AUC values can be seen in Table 4.

## 4. Discussion

This is the first study to reveal detailed differences in temporal aspects of language processing between alcohol-dependent patients and healthy individuals. Our major finding is that the filled pause-related temporal parameters appear to be particularly altered in ARCI. Since 10 out of the examined 15 TSPs significantly differed between AUD patients and healthy controls, we suggest that TSPs measured by the S-GAP Test^®^ may be a promising tool for the early recognition of cognitive impairment in AUD.

Although the effect of alcohol use on higher-order language functions has been observed [42], to the best of our knowledge, TSPs had not previously been studied in AUD patients.

In the present study, the comparison of the recordings of the AUD and control groups showed significant differences in 10 of the 15 evaluated TSPs, with the greatest differences in the speech tempo, filled pause occurrence rate, total pause occurrence rate and silent pause average duration. Apart from the difference in speech tempo, the findings of significant differences in pauses in speech are in accordance with earlier TSP results in Alzheimer’s disease (AD) and related mild cognitive impairment (MCI) [28,31,43], and with other speech analysis methods [44,45,46]. These results seem to indicate that these pauses in speech production could show early signs of cognitive deficits in AUD as well. These observed, subtle cognitive changes may be explained by the complex neurobiological effect of AUD.

Similarly to our other S-GAP-related studies in neurocognitive disorders [28,31,43], we also found that several TSPs have classification potential in the differentiation between cognitively intact AUD patients and healthy controls. Our results indicate that subtle temporal speech-related parameters are present in the early stage of AUD. This also shows the importance of speech-related pauses in detecting cognitive changes.

The difference between the AUD patients and healthy controls was most prominent in the case of the speech tempo, filled pause occurrence rate, total pause duration rate and silent pause average duration, which suggests that these TSPs may be specific in ARCI. These anomalies in language production are connected to several higher cognitive functions, especially to mental load and processing speed [28,31,46].

The main novelty of our study is that these linguistic representations can indicate early cognitive decline in patients with AUD that cannot be detected using available simple neurocognitive tests such as the MMSE. The early detection of ARCI is of utmost importance in this patient group, since cognitive deficits affect patients’ daily functioning and adherence to treatment [47] and could increase their relapse rate [48]. Given these factors, ARCI could affect the risk of developing lethal complications (e.g., delirium tremens), deteriorate patients’ quality of life and shorten their life expectancy.

Our previous findings showed that the TSPs can also discriminate patients with mild cognitive impairment (MCI) from healthy individuals with mild AD [29,31,33,49]. Our previous results revealed that the utterance length, silent pause duration rate, total pause duration rate, silent pause mean duration and total pause mean duration showed significant differences, and the speech tempo and total pause number were the most sensitive parameters for differentiating MCI from controls [31,33]. Both our present findings in AUD and our former results in MCI and AD patients indicate that subtle speech abnormalities are not specific to a nosologic category.

Our major findings reflect that dysfunctional language production in ARCI could be an early sign of subtle neurobiological alterations due to the long-term consequences of alcohol consumption. Disruption in inhibitory control is among the most important psychobiological features of various forms of addictions, including AUD [50,51,52]. The frontostriatal circuit is involved in language processing [53]. The frontostriatal circuit is primarily involved in the regulation of inhibitory functions; therefore, disturbance of this loop contributes to the impulsive and compulsive features of substance use disorders [54,55]. Besides the frontostriatal circuit the superior longitudinal fasciculus and arcuate fasciculus are important components of the complex language networks, as confirmed by connectome studies [56].

In summary this study is the first to reveal detailed differences in the temporal aspect of language production between alcohol-dependent patients in the early stage of abstinence and healthy individuals. The results of our exploratory study reveal that filled pause-related temporal parameters appear to be particularly altered in ARCI compared with other cognitive disorders. The findings of our work suggest that certain TSPs are sensitive indicators of early cognitive impairment in AUD.

The major limitation of our work is the small number of participants. The next concern is that the severity of AUD was only determined using the AUDIT. Furthermore, we were not able to explore correlations between different cognitive domains and the linguistic representations since we were not using a comprehensive neuropsychological test battery for cognitive assessment. Finally, the cross-sectional nature of our study design also limits the generalizability of our findings.

Longitudinal prospective case–control studies are required in larger populations in order to validate our findings and to examine the changes in cognition in the different stages of abstinence.

## Figures and Tables

**Table 1:** List and definitions of the temporal parameters of spontaneous speech.

Temporal Speech Parameter	Description
Utterance length (s)	Total duration of the utterance
Articulation tempo (phone/s)	The number of phones in one second in the total length of the utterance without (silent and filled) pauses
Speech tempo (phone/s)	The number of phones in one second in the total length of the utterance including (silent and filled) pauses
Silent pause occurrence rate (%)	Total number of silent pauses (count) × 100/total number of phones (count)
Filled pause occurrence rate (%)	Total number of filled pauses (count) × 100/total number of phones (count)
Total pause occurrence rate (%)	Total number of silent and filled pauses (count) × 100/total number of phones (count)
Silent pause duration rate (%)	Total length of silent pauses (s) × 100/total length of the utterance (s)
Filled pause duration rate (%)	Total length of filled pauses (s) × 100/total length of the utterance (s)
Total pause duration rate (%)	Total length of silent and filled pauses (s) × 100/total length of the utterance (s)
Silent pause frequency (1/s)	Total number of silent pauses (count)/total length of the utterance (s)
Filled pause frequency (1/s)	Total number of filled pauses (count)/total length of the utterance (s)
Total pause frequency (1/s)	Total number of silent and filled pauses (count)/total length of the utterance (s)
Silent pause average duration (s)	Total length of silent pauses (s)/total number of silent pauses (count)
Filled pause average duration (s)	Total length of filled pauses (s)/total number of filled pauses (count)
Total pause average duration (s)	Total length of silent and filled pauses (s)/total number of silent and filled pauses (count)

**Table 2:** Clinical characteristics of participants and comparison of the results of the psychometric tests between the AUD and control groups.

	Control Group (*n* = 31)		AUD Group (*n* = 34)		
	M	*SD*	M	*SD*	*t*-Test, Significance
Mean age	9.795	50.71	9.39	48.35	
AUDIT	2	1.746	26	8.582	z = (−7.011), ***p* < 0.001**
AUDIT-C	2	1.675	11	2.375	z = (−6.773), ***p* < 0.001**
AUDIT-D	0	0	7.50	3.798	z = (−6.771), ***p* < 0.001**
AUDIT-HE	0	0.359	5.50	3.713	z = (−7.124), ***p* < 0.001**

Abbreviations: AUDIT = Alcohol Use Disorders Identification Test, AUDIT-C = Alcohol Use Disorders Identification Test Consumption subscale, AUDIT-D = Alcohol Use Disorders Identification Test Dependence subscale, AUDIT-HE = Alcohol Use Disorders Identification Test Harmful Effects subscale; AUD = alcohol dependency syndrome, M = median, *SD* = standard deviation.

**Table 3:** Comparison of temporal speech parameters between the AUD and control groups.

	Control Group (*n* = 31)		AUD Group (*n* = 34)		*t*-Test, Significance	Cohen’s d
	M	*SD*	M	*SD*		
Articulation tempo	14.743	1.544	14.551	1.186	*t*(63) = 0.567, *p* = 0.573	−0.141
Speech tempo	9.647	1.551	7.877	2.038	*t*(63) = 3.912, ***p* < 0.001**	−0.971
Utterance length	84.052	34.760	77.569	44.481	*t*(63) = 0.650, *p* = 0.518	−0.161
Silent pause occurrence rate	4.850	1.628	5.243	1.907	*t*(63) = −0.889, *p* = 0.378	0.221
Filled pause occurrence rate	1.512	0.897	2.831	1.695	*t*(51.083) = −3.970, ***p* < 0.001**	0.960
Total pause occurrence rate	6.362	1.935	8.074	2.491	*t*(63) = −3.073, ***p* = 0.003**	0.763
Silent pause duration rate	30.538	9.739	39.112	15.862	*t*(55.494) = −2.651, ***p* = 0.010**	0.644
Filled pause duration rate	3.798	2.618	6.914	5.704	*t*(47.235) = −2.871, ***p* = 0.006**	0.692
Total pause duration rate	34.336	9.568	46.025	12.984	*t*(63) = −4.098, ***p* < 0.001**	1.018
Silent pause frequency	0.480	0.115	0.419	0.113	*t*(63) = 2.177, ***p* = 0.033**	−0.541
Filled pause frequency	0.154	0.087	0.245	0.155	*t*(52.661) = −2.943, ***p* = 0.005**	0.713
Total pause frequency	0.634	0.131	0.663	0.196	*t*(58.040) = −0.709, *p* = 0.481	0.173
Silent pause average duration	0.641	0.155	0.983	0.488	*t*(40.161) = −3.877, ***p* < 0.001**	0.927
Filled pause average duration	0.243	0.078	0.246	0.090	*t*(63) = −0.163, *p* = 0.871	0.041
Total pause average duration	0.550	0.142	0.775	0.406	*t*(41.610) = −3.033, ***p* = 0.004**	0.726

Abbreviations: AUD = alcohol use disorder, M = mean, *SD* = standard deviation.

**Table 4:** ROC analysis and Area Under the Curve of temporal speech parameters with the highest classification potential.

Area Under the Curve			
Temporal Speech Parameter	Area	*SD*	Asymptotic Significance
Filled pause occurrence rate	0.795	0.057	0.000
Total pause occurrence rate	0.714	0.067	0.003
Filled pause duration rate	0.720	0.071	0.002
Total pause duration rate	0.732	0.066	0.001
Filled pause frequency	0.723	0.066	0.002
Silent pause average duration	0.673	0.070	0.016

Abbreviation: *SD* = standard deviation.

## Data Availability

The dataset from this study is available from the corresponding author (János Kálmán) upon request. This anonymized dataset was generated from the registered official health insurance patient flow of the university clinic, and due to the official data protection policy, this data is preferred to be made available not fully but only on request. Further enquiries can be directed to the corresponding author (János Kálmán).

## Abbreviations

The following abbreviations are used in this manuscript:

AD	Alzheimer’s disease
ARCI	Alcohol-related cognitive impairment
ASR	Automatic speech recognition
AUD	Alcohol use disorder
AUDIT	Alcohol Use Disorders Identification Test
AUDIT-C	Alcohol Use Disorders Identification Test Consumption subscale
AUDIT-D	Alcohol Use Disorders Identification Test Dependence subscale
AUDIT-HE	Alcohol Use Disorders Identification Test Harmful Effects subscale
CIWA-Ar	Clinical Institute Withdrawal Assessment Alcohol Scale Revised
DNN	Deep Neural Network
GBD	Global Burden of Disease
HTK	Hidden Markov Model Tool Kit
ICD-10	International Classification of Diseases, Tenth Revision
M	Mean
MMSE	Mini-Mental State Examination
ROC	Receiver operating characteristic
SD	Standard deviation
S-GAP	Speech-GAP Test
TSP	Temporal speech parameter
WHO	World Health Organization
